# Supplementary material for: Deletion of wfs1 Impairs Oligodendrocyte Precursor Cells Dorsal Distribution and Myelination Through the wfs1-hmgcs1 Axis in Zebrafish
Source: Biology (Basel). 2026 Aug 16;15(16):1402. doi: 10.3390/biology15161402 (PMC13510151; doi:10.3390/biology15161402)
Supplement: Supplementary file 1 [file biology-15-01402-s001.zip › File S1-Transcriptome sequencing data and differentially expressed genes (DEGs) in wfs1-- zebrafish at 5 dpf/2.KEGG/wfs1KOvsWT/UP/src/dre04144.html]

dre04144


- dre:406837

- Up regulated genes

406837\_1(6.87422374888232)
- dre:556277

- Up regulated genes

556277(2.56614816386233)

- dre:336368

- Up regulated genes

336368(6.40118454252161)

- dre:573376

- Up regulated genes

573376(2.09344333483373)

- dre:336426

- Up regulated genes

336426(1.55368624578632)

- dre:793158

- Up regulated genes

793158\_1(1.86160461223548)
- dre:556226

- Up regulated genes

556226\_1(6.36622858283894)

- dre:792828

- Up regulated genes

792828\_1(7.34573383982123)

- dre:568170

- Up regulated genes

568170\_1(1.60786733087771)

- dre:245702

- Up regulated genes

245702(1.27755220735994)

- dre:792229

- Up regulated genes

792229\_1(2.08412818777288)

- dre:565743

- Up regulated genes

565743\_1(2.40489825782141)

- dre:565743

- Up regulated genes

565743\_1(2.40489825782141)

- dre:561771

- Up regulated genes

561771(1.57208181397583)

- dre:436718

- Up regulated genes

436718(1.34229427553561)

- dre:556277

- Up regulated genes

556277(2.56614816386233)

- dre:565743

- Up regulated genes

565743\_1(2.40489825782141)

- dre:474327

- Up regulated genes

474327(1.18169964792452)

- dre:406684

- Up regulated genes

406684\_1(1.82758055327184)
- dre:393881

- Up regulated genes

393881(1.10381479961769)

Close
